# Effect of Intravenous Vitamin C, Thiamine, and Hydrocortisone (The Metabolic Resuscitation Protocol) on Early Weaning from Vasopressors in Patients with Septic Shock. A Descriptive Case Series Study

**DOI:** 10.7759/cureus.5016

**Published:** 2019-06-27

**Authors:** Hassan Masood, Ahmed M Burki, Anum Sultan, Hina Sharif, Asim Ghauri, Sehrish Khan, Muhammad Shoaib Safdar Qureshi, Aayesha Qadeer, Ghulam Rasheed

**Affiliations:** 1 Internal Medicine, Shifa International Hospital, Islamabad, PAK; 2 Anesthesiology and Critical Care, Combined Military Hospital, Rawalpindi, PAK; 3 Anesthesiology, Combined Military Hospital, Rawalpindi, PAK; 4 Public Health, Al-Shifa School of Public Health, Islamabad, PAK; 5 Ophthalmology, Al-Shifa Trust Eye Hospital, Rawalpindi, PAK

**Keywords:** septic shock, vitamin c, hydrocortisone, vasopressors, thiamine

## Abstract

Objectives: The aim of this study was to assess the efficacy of intravenous vitamin C, hydrocortisone, and thiamine in early weaning (within 48 hours) from vasopressor support in patients with septic shock. We also aimed to assess mortality and intensive care unit (ICU) stay.

Study Design: We conducted a descriptive case series study of 50 patients with septic shock who were admitted in the ICU of the Combined Military Hospital Rawalpindi in Pakistan from August 2017 until April 2018.

Materials and Methods: The study included men and women (16 to 80 years of age) who were admitted to the ICU with septic shock. Data were analysed using the IBM Statistical Package for Social Sciences (SPSS), version 18.0 (IBM Corp., Armonk, NY, USA). Inferential analysis was done with the help of simple and multivariate binary logistic regression that generated unadjusted and adjusted odds ratios (OR), respectively.

Results: Of the 50 patients, 56% (N = 28) were male with a mean age of the respondents being 46.7 ± 18.4. Eighty-four percent were successfully weaned off vasopressors within 48 hours. Median days in the ICU were reported as 8.3 (interquartile range (IQR) = 5). Primary bacteremia (34%) was the most reported cause of ICU admission. The most common vasopressor was norepinephrine and its mean dose was 21.6 ± 10.7 microgram/min. The ICU mortality was observed at 52% (N = 26). Unadjusted OR showed a dose of norepinephrine, Sequential Organ Failure Assessment (SOFA) score, plasma procalcitonin, and plasma lactate to be significant predictors (p-value < 0.05), while the adjusted odds ratio (AOR) showed only a dose of norepinephrine to be a statistically significant predictor (AOR = 0.804, 95% CI = 0.674 - 0.960; p-value = 0.016).

Conclusion: The administration of intravenous vitamin C, hydrocortisone, and thiamine to patients with septic shock was successful in early weaning from vasopressors. There was also a reduction in procalcitonin and lactate levels, as well as the SOFA score. Further trials are needed to determine whether the metabolic resuscitation protocol can become part of the treatment for septic shock.

## Introduction

Sepsis is among one of the leading causes of morbidity and mortality worldwide, accounting for nearly half of all admissions to intensive care units. It has a global burden of 15 to 20 million cases annually. In low-income countries, this roughly accounts for 60% of mortality [1].

Sepsis is defined as life-threatening organ dysfunction caused by a dysregulated host response to infection. On the other hand, septic shock is defined as a subset of sepsis in which underlying circulatory and cellular metabolism abnormalities are profound enough to substantially increase mortality [2]. Apart from its immediate dangers, such as multisystem organ dysfunction, sepsis patients also have increased two-year mortality approaching 45% and a low quality of life [3].

Despite the advances made in identifying and treating patients with sepsis, the advent of newer antibiotics with broader coverage, and an aggressive guidelines strategy of the surviving sepsis campaign, the morbidity and mortality associated with sepsis remain high. As of yet, no novel therapeutic intervention has been found that has been proven to improve the outcome in sepsis.

We had a patient in the Intensive Care Unit (ICU) who had septic shock requiring three vasopressors. We decided to administer vitamin C, thiamine, and hydrocortisone to this patient on the basis of emerging clinical data. The patient improved dramatically and was off vasopressors in less than 24 hours. He was shifted out of the ICU seven days later. He was later discharged from the hospital with no residual organ dysfunction.

Mitochondrial dysfunction occurs early in sepsis and is considered to be one of the key pathological phenomenon involved in sepsis [4]. In sepsis, there is impaired perfusion at the organ level which leads to insufficient oxygen at the mitochondrial level to drive oxidative phosphorylation of adenosine diphosphate (ADP) to adenosine triphosphate (ATP) [5].

The ascorbic acid concentration in plasma and leukocytes is found to be subnormal in patients with sepsis, and there is an inverse relation between ascorbic acid levels and the incidence of multisystem organ failure [6-7]. These patients require a high-dose intravenous administration of vitamin C of up to 6.0 grams per day for correction [8].

Marik et al. reported that the early administration of intravenous vitamin C, in combination with thiamine and steroids, was effective in reducing the mean duration of vasopressors in addition to reducing mortality and preventing progressive organ dysfunction in patients with severe sepsis and septic shock [9].

To this researcher’s best knowledge, there was no locally available data in Pakistan on the use of the metabolic resuscitation protocol in sepsis. This study aims to assess the efficacy of the combination of intravenous vitamin C, thiamine, and hydrocortisone in terms of early weaning (within 48 hours) from vasopressor support, ICU mortality, and the length of ICU stay in patients with septic shock.

## Materials and methods

We conducted a descriptive case series study in ICU of the Combined Military Hospital (CMH), Rawalpindi. After approval from the hospital ethical review committee, 50 patients with septic shock were selected by non-probability convenient sampling. They were started on the metabolic resuscitation cocktail which included intravenous vitamin C, 1,500 mg every six hours, intravenous hydrocortisone, 50 mg every six hours, and intravenous thiamine, 200 mg every 12 hours. This treatment was continued for five days, in addition to the routine treatment of septic shock. Data were collected on individual proformas and were analyzed using the IBM Statistical Package for Social Sciences (SPSS), version 18.0 (IBM Corp., Armonk, NY, USA). Daily C-reactive protein (CRP), lactate, and plasma procalcitonin levels were checked. The Sequential Organ Failure Assessment (SOFA) score was also calculated on a daily basis. Pregnant women or patients already on vasopressor support for more than 24 hours were excluded from the study. The data collection tool (proforma) was comprised of two sections: demographic and clinical variables. Variables were both quantitative and categorical in nature. Descriptive analysis was done with the help of mean ± standard deviation, median with an interquartile range (IQR) (where relevant) for quantitative variables. For categorical variables, frequencies with the percentage were run. Inferential analysis was conducted in two steps. In the first step, univariate binary logistic regression was done to screen out significant unadjusted odds ratio with Wald statistics and unstandardized beta. In the next step, multivariate binary logistic regression was run with all significant variables from univariate analysis to identify significant predictors with adjusted odds ratio (AOR) and 95% confidence interval.

## Results

Descriptive results are shown in Tables 1-2. This cross-sectional study observed a 52% (N = 26) mortality. Fifty-six percent (N = 28) were male, and the mean age of respondents was 46.7 ± 18.4 (range: 16 to 77 years old), while the median days in the ICU were reported as 8.3 (IQR = 5). The average temperature of respondents was observed as 101.5 ± 10.7 ºF, and primary bacteremia (34%) was the most reported cause of the ICU admission. The mean arterial blood pressure in mmHg (47.7 ± 6.17) had a minimum value of 25 and a maximum of 58 units. The most common vasopressor was norepinephrine; a combination of norepinephrine and epinephrine was administered to 30% of patients, while the addition of dobutamine, an inotropic agent, was observed for 4% of patients (N = 2). The mean dose of norepinephrine was 21.6 ± 10.7 micrograms/min, and it was 9.7 ± 3.3 micrograms/min for epinephrine. Plasma procalcitonin ranged from 2.20 to 30 with a mean reading of 7.73 ± 5.73, while for plasma lactate (4.4 ± 2.3), a minimum of 1.56 and a maximum of 15 units were observed.

**Table 1:** Descriptive for Continuous Variables CRP: C-reactive protein; SD: standard deviation; SOFA: Sequential Organ Failure Assessment

Variable	Mean ± SD	Min - Max
Mean arterial blood pressure	47.7 ± 6.2	25 - 58
Heart rate	123.4 ± 22.7	52 - 161
SOFA score	8.5 ± 1.5	6 - 12
Plasma procalcitonin	7.7 ± 5.7	2 - 30
Plasma lactate	4.4 ± 2.3	1.6 - 15
CRP	177.4 ± 51.9	92 - 310

**Table 2:** Descriptive for Categorical Variables

Variables	% (N)
Gender	
Male	56 (28)
Female	44 (22)
Diagnosis	
Pneumonia	20 (10)
GI/biliary disease	30 (15)
Primary bacteremia	34 (17)
Others	16 (8)
Vasopressor medicine	
Norepinephrine	66 (33)
Norepinephrine + epinephrine	30 (15)
Norepinephrine + epinephrine + dobutamine	4 (2)
Vasopressor suspension (within 48 hours)	
No	16 (8)
Yes	84 (42)
Duration of vasopressor support	
< 24 hours	58 (29)
24 - 48 hours	26 (13)
Mortality	
No	52 (26)
Yes	48 (24)

**Figure 1:**
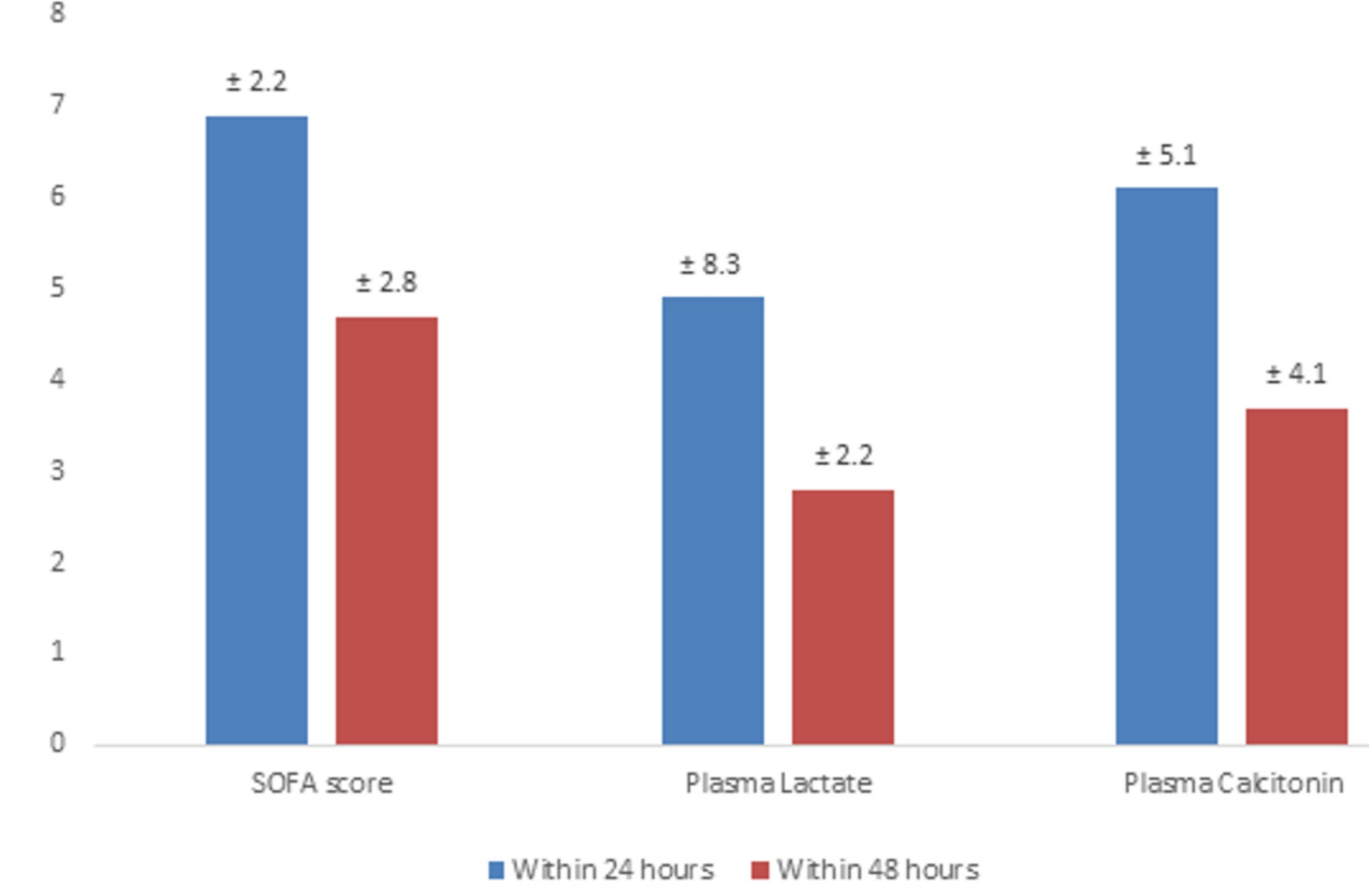
Trend of SOFA score, plasma lactate, and plasma calcitonin in the ICU ICU: Intensive Care Unit; SOFA: Sequential Organ Failure Assessment

The vasopressor was suspended within 48 hours for eight patients (16%), while for 29 patients (48%), it was suspended within 24 hours. The mean CRP within 24 and 48 hours was 149 ± 59.0 and 115.2 ± 58.3, respectively; other readings are listed in Figure 1.

Inferential

The results from the univariate binary logistic regression showed a dose of norepinephrine, SOFA score, plasma procalcitonin, and plasma lactate to be a significant predictor of mortality (p-value < 0.05) (Table 3). The unstandardized beta (-0.188) for the dose of norepinephrine showed that the greater the dose of this vasopressor, the less probability of suspending vasopressor within 48 hours, while the AOR = 0.828 (95% CI = 0.735 - 0.934) also showed the less likelihood of suspending vasopressor for patients with every 1 microgram/min dose. Female patients had less chance (unadjusted OR = 0.75, 95% CI = 0.16-3.41) of vasopressor duration to end within 48 hours as compared to male patients but it was not statistically significant (p-value = 0.71). The findings further revealed that the higher the reading for plasma procalcitonin, the less will be the probability of halting vasopressor support (unstandardized beta: -2.11); the unadjusted odds ratio (AOR) showed 0.810 less likelihood of outcome with a higher reading of this predictor, and it was statistically significant (p-value = 0.007).

**Table 3:** Univariate Binary Logistic Regression * indicates the value is statistically significant CI: confidence interval; CRP: C-reactive protein; ICU: Intensive Care Unit; OR: odds ratio; SOFA: Sequential Organ Failure Assessment

Variable	Unadjusted OR	95% CI	Unstandardized Beta	Wald Statistic	p-value
Age	1.00	0.96 - 1.04	0.004	0.03	0.85
Gender					
Male	1	--	--	0.14	0.71
Female	0.75	0.16 - 3.41	--	--	--
Days in ICU	1.55	0.95 - 2.52	0.438	3.09	0.078
Dose of epinephrine (microgram/min)	0.947	0.70 - 1.28	-0.055	0.127	0.721
Dose of norepinephrine (microgram/min	0.828	0.735 - 0.934	-0.188	9.50	0.002*
Mean arterial blood pressure	1.094	0.976 - 1.225	0.090	2.39	0.121
Heart rate	0.975	0.936 - 1.02	-0.025	1.43	0.232
SOFA score	0.60	0.352 - 1.02	-0.512	3.55	0.040*
Plasma procalcitonin	0.810	0.695 - 0.944	-2.11	7.24	0.007*
Plasma lactate	0.511	0.303 - 0.862	-0.67	6.34	0.012*
CRP	0.992	0.978 - 1.002	-0.008	1.25	0.263

There was an indirect relation between the reading of plasma lactate and outcome (unstandardized beta: -0.67); with a higher reading of plasma lactate, there was less chance (unadjusted OR = 0.51) of suspending vasopressor within 48 hours, and it was statistically significant (p-value = 0.012).

The AOR from multivariate binary logistic regression showed only the dose of norepinephrine to be statistically significantly associated with the outcome (Table 4). For every one unit increase in the dose (microgram/min) of norepinephrine, there was less chance of suspending vasopressor within 48 hours, even after controlling for other factors (AOR = 0.804, 95% CI = 0.674 - 0.960). 

**Table 4:** Multivariate Logistic Regression * indicates the value is statistically significant AOR: adjusted odds ratio; CI: confidence interval; SOFA: Sequential Organ Failure Assessment

Variable	AOR	95% CI	p-value
Dose of norepinephrine (microgram/min)	0.804	0.674 - 0.960	0.016*
SOFA score	2.53	0.769 - 8.293	0.127
Plasma procalcitonin	0.88	0.681 - 1.16	0.378
Plasma lactate	0.616	0.29 - 1.33	0.217

## Discussion

Sepsis is a highly morbid entity and a number of interventions have been tried to decrease the morbidity and mortality associated with this. The highest mortality is seen in developing countries where its 28-day mortality is 60% as compared to 25% in developed countries [9-11]. There are many other short and long-term complications associated with sepsis, as well as associated with pharmacologic agents [12].

In the present study, vasopressor support was suspended within 48 hours for eight patients (16%), while for 29 patients (48%), it was suspended within 24 hours. These results were supported by a number of studies done in the past which have shown that this combination has rapidly decreased the required doses of vasopressors in cases with sepsis [9, 13-14].

According to a study done by Marik et al., they compared the same protocol with controls and it was seen that vasopressors were weaned off in all the subjects; the mean time for weaning was 18.3 ± 9.8 hours after augmentation of this treatment. In the control group, this was seen in 54.9 ± 28.4 hours with a p-value of < 0.001. Furthermore, they found the mortality to be 9% in the treatment group as compared to 41% in the control group [9].

In another study carried out by Kim et al. where they compared this combination for the treatment of severe pneumonia, it was seen that there was significant mortality benefit of 17% vs 39% with a p-value of 0.04 [13]. Moskowitz et al. also supported the results of the above-mentioned study by elaborating the utility of this combination at the molecular level in detail [14].

This was similar to the present study where plasma procalcitonin and plasma lactate were significant predictors regarding vasopressors (p-value < 0.05). The findings further revealed that the higher the reading for plasma procalcitonin, the less will be the probability of halting vasopressor support (unstandardized beta: -2.11), while the unadjusted odds ratio showed 0.810 less likelihood of outcome with a higher reading of this predictor and it was statistically significant (p-value = 0.007).

There are a number of studies and trials going on using a single agent and a combination of these two and three agents. Steroids have shown very promising results in the past and have shown efficacy in terms of early weaning from ionotropic support, ventilator-free days, and the time it takes to recover from the shock. Similarly, in the Corticosteroid Therapy of Septic Shock (CORTICUS) trial where steroids were administered as a single add-on agent in sepsis, the mean time for weaning vasopressors was 79 hours which was far higher as compared to within 24 hours in the present study [16-17].

The basic concept of the conjunction of ascorbic acid and steroids is that the ascorbic acid increased the number of glucocorticoid receptors which are dysfunctioned due to oxidising molecules in sepsis. On the other hand, steroids also increased the cellular uptake for vitamin C via sodium vitamin C transporter channel [18].

According to a study done by Fowler et al., the minimum vasopressor time needed to wean after ascorbic acid (vitamin C) replacement in sepsis was 86 hours [18]. This can further benefit the unwanted side effects that were observed with the longer use of norepinephrine which were due to its anti-inflammatory and bacteria growth-promoting effects [19]. The studies carried out by Tanaka et al. and Long et al. found a great benefit of vitamin C use in enhancing endothelial function and microcirculatory flow [20-21].

Our study was limited in that we had a small sample size. We were also unable to do random sampling which limited the generalizability of our study results. Further randomized control studies with a larger patient population group are warranted to confirm our results

## Conclusions

The combined administration of intravenous vitamin C, hydrocortisone, and thiamine to patients with septic shock was successful in the early weaning from vasopressors in patients with septic shock. There was also a reduction in procalcitonin and lactate levels, as well as the SOFA score. Given the potential benefits of the metabolic resuscitation protocol, critical care physicians should consider its use in patients with septic shock.

## References

[1] Adhikari NK, Fowler RA, Bhagwanjee S, Rubenfeld GD (2010). Critical care and the global burden of critical illness in adults. Lancet.

[2] Singer M, Deutschman CS, Seymour CW (2016). The Third International Consensus Definitions for Sepsis and Septic Shock (Sepsis-3). JAMA.

[3] Karlsson S, Ruokonen E, Varpula T, Ala-Kokko TI, Pettilä V, Finnsepsis Study Group (2009). Long-term outcome and quality-adjusted life years after severe sepsis. Crit Care Med.

[4] Singer M (2014). The role of mitochondrial dysfunction in sepsis-induced multi-organ failure. Virulence.

[5] Stidwill RP, Rosser DM, Singer M (1998). Cardiorespiratory, tissue oxygen and hepatic NADH responses to graded hypoxia. Intensive Care Med.

[6] Schorah CJ, Downing C, Piripitsi A, Gallivan L, Al-Hazaa AH, Sanderson MJ, Bodenham A (1996). Total vitamin C, ascorbic acid, and dehydroascorbic acid concentrations in plasma of critically ill patients. Am J Clin Nutr.

[7] Borrelli E, Roux-Lombard P, Grau GE, Girardin E, Ricou B, Dayer JM, Suter PM (1996). Plasma concentrations of cytokines, their soluble receptors, and antioxidant vitamins can predict the development of multiple organ failure in patients at risk. Crit Care Med.

[8] Padayatty SJ, Sun H, Wang Y (2004). Vitamin C pharmacokinetics: implications for oral and intravenous use. Ann Intern Med.

[9] Marik PE, Khangoora V, Rivera R, Hooper MH, Catravas J (2017). Hydrocortisone, vitamin C, and thiamine for the treatment of severe sepsis and septic shock: a retrospective before-after study. Chest.

[10] Kaukonen KM, Bailey M, Suzuki S, Pilcher D, Bellomo R (2014). Mortality related to severe sepsis and septic shock among critically ill patients in Australia and New Zealand, 2000-2012. JAMA.

[11] Kadri SS, Rhee C, Strich JR (2017). Estimating ten-year trends in septic shock incidence and mortality in United States academic medical centers using clinical data. Chest.

[12] Gaieski DF, Edwards JM, Kallan MJ, Carr BG (2013). Benchmarking the incidence and mortality of severe sepsis in the United States. Crit Care Med.

[13] Kim W, Jo E, Eom JS (2018). Combined vitamin C, hydrocortisone, and thiamine therapy for patients with severe pneumonia who were admitted to the intensive care unit: propensity score-based analysis of a before-after cohort study. J Crit Care.

[14] Moskowitz A, Andersen LW, Huang DT (2018). Ascorbic acid, corticosteroids, and thiamine in sepsis: a review of the biologic rationale and the present state of clinical evaluation. Crit Care.

[15] Balakrishnan M, Gandhi H, Shah K, Pandya H, Patel R, Keshwani S, Yadav N (2018). Hydrocortisone, vitamin C and thiamine for the treatment of sepsis and septic shock following cardiac surgery. Indian J Anaesth.

[16] Sprung CL, Annane D, Keh D (2008). Hydrocortisone therapy for patients with septic shock. N Engl J Med.

[17] Marik PE (2011). Glucocorticoids in sepsis: dissecting facts from fiction. Crit Care.

[18] Fisher BJ, Seropian IM, Kraskauskas D, Thakkar JN, Voelkel NF, Fowler AA 3rd, Natarajan R (2011). Ascorbic acid attenuates lipopolysaccharide-induced acute lung injury. Crit Care Med.

[19] Fowler AA 3rd, Syed AA, Knowlson S (2014). Phase 1 safety trial of intravenous ascorbic acid in patients with severe sepsis. J Transl Med.

[20] Tanaka H, Matsuda T, Miyagantani Y, Yukioka T, Matsuda H, Shimazaki S (2000). Reduction of resuscitation fluid volumes in severely burned patients using ascorbic acid administration: a randomized, prospective study. Arch Surg.

[21] Long CL, Maull KL, Krishman RS (2003). Ascorbic acid dynamics in the seriously ill and injured. J Surg Res.

